# The impact of vaccine hesitation on the intentions to get COVID-19 vaccines: The use of the health belief model and the theory of planned behavior model

**DOI:** 10.3389/fpubh.2022.882909

**Published:** 2022-10-14

**Authors:** Zeming Li, Ying Ji, Xinying Sun

**Affiliations:** Department of Social Medicine and Health Education, School of Public Health, Peking University, Beijing, China

**Keywords:** vaccination intention, COVID-19, TPB (theory of planned behavior), HBM (health belief model), vaccine hesitation

## Abstract

**Object:**

During the later period of the COVID-19 pandemic, the public has been at risk of the evolving COVID-19 variants and hesitated to be vaccinated against COVID-19 to a certain extent. In this context, the health belief model (HBM) and the theory of planned behavior model (TPB) were used to compare and summarize the relationship between vaccine hesitation/non-hesitation and the intentions to get COVID-19 vaccines and its influencing factors.

**Methods:**

The cross-sectional, population-based online survey was conducted from 14 April to 30 April 2021, and 1757 respondents were recruited to participate in the survey through the Wenjuanxing online survey platform. The HBM and TPB covariate scores were expressed using means and standard deviations and compared between groups using t-tests. Backward multiple linear regression models were used to explore the factors influencing the public's intentions to receive the COVID-19 vaccines.

**Results:**

This study found that educational background is one of the factors influencing vaccine hesitation. Most people with high education do not hesitate (65.24%), while a more significant proportion of people with low education have vaccine hesitation (66.00%). According to HBM, for the vaccine hesitation group, self-efficacy, family advice, and doctor's advice were the most critical factors affecting the public's future vaccination intentions; for the vaccine non-hesitation group, self-efficacy, doctor's advice, and perceived benefits are the most important influencing factors. According to the TPB, the subjective norm is the most critical factor affecting the future vaccination intention of the vaccine hesitation group, and the attitude toward behavior is the most critical factor affecting the future vaccination intention of the vaccine non-hesitation group.

**Conclusions:**

In the context of COVID-19, the public's hesitation on the “current” vaccines will still affect future vaccination intentions. Using HBM and TPB would help health policymakers and healthcare providers formulate intervention plans.

## Introduction

COVID-19 is a fast-growing disease that spreads globally (1, 2), which seriously endangers human health and causes significant damage to social and economic development. Global public health practices have demonstrated that vaccination is one of the safest and most effective measures to prevent control and infectious diseases (3, 4). Since December 2020, the COVID-19 vaccine has been used in some countries. However, not everyone is willing to be vaccinated due to the relatively short development time of the COVID-19 vaccine, misleading information, negative emotion, easy access to the vaccine, and concern about vaccine safety (5, 6). As a result, a phenomenon of vaccine hesitation exists, that is, to refuse or delay the vaccination for COVID-19. Vaccine hesitation was proposed in 2012 by the World Health Organization Strategic Advisory Group of Experts (SAGE), meaning that “the public continues to delay or refuse vaccination despite available vaccination service” (7). In an early April 2020 study of 991 U.S. adults assessing attitudes toward COVID-19 vaccines, Fisher et al. found that 31.6% were unsure about getting vaccinated, and 10.8% were not planning to get vaccinated (5). Lazarus et al. studied 13,426 randomly selected individuals in 19 countries for possible COVID-19 vaccines, of which 71.5% responded that they would be vaccinated if proven safe and effective, and 61.4% of people said they would get vaccinated if their employer recommended it (8). LIU et al. surveyed 2,531 Chinese adults, of which 44.3% were hesitant about vaccines (9). Vaccine hesitation is one of the world's top ten health threats and has a strong negative relationship with the vaccination rate. It impacts public acceptance of the vaccine (10, 11), thus reducing the vaccination rate and herd immunity, weakening the vaccine's protective effect, increasing the outbreak and epidemic of vaccine-preventable diseases, and threatening public health (12, 13).

At the same time, virus variation is one of living beings' natural characteristics. The majority of viruses vary over time, and the potential for viruses to mutate increases as the number of people infected increases. For example, the variant Omicron of the COVID-19 found in South Africa in November 2021 is spreading worldwide at tremendous speed, leading to a new peak of cases and deaths, which has become the dominant strain worldwide (14). In response, the WHO experts suggest that regular vaccination against COVID-19 may be required in the future (15). The theoretical model related to health belief and risk perception is an essential tool for understanding the factors underlying behavioral decision-making by assessing factors that motivate or inhibit the public from adopting health-related behavior (16). Theoretical models such as the Health Belief Model (HBM) and the Theory of Planned Behavior model (TPB) indicate that behavioral intention is essential for individuals to produce healthy behaviors. Therefore, theoretical models can be used to explore and explain the influencing factors of the public's intentions to get the COVID-19 vaccines from a social and psychological perspective.

Health Belief Model (HBM) is one of the models widely used to understand health or disease behavior (17). Based on the HBM, factors influencing the public's future willingness to be vaccinated against COVID-19 include that.

1) Perceived severity is the perception of the severity and consequences of individuals or others when infected with the virus;

2) Perceived susceptibility is the perception of the risk possibility of contracting the virus;

3) Perceived benefits are the perception of the potential advantages brought by vaccination (such as reducing the risk of infection);

4) Perceived barriers are the perception of the difficulties caused by vaccination (including physical ones, such as side effects, and both psychological and financial ones);

5) Self-efficacy is an individual's evaluation and judgment of the ability to solve the problems resulting from vaccination;

6) Cues to action are the 'triggers' that inspire or evoke the actor to take action.

Previous studies have shown that the structure of HBM is an essential predictor of influenza vaccination (18–21). Wong LP et al. assessed the acceptability and willingness to pay (WTP) of COVID-19 vaccines using the Health Belief Model (22), and Wong MCS et al. predicted the acceptance of a COVID-19 vaccine among Hong Kong adults using the Health Belief Model and found that the government recommendation was a significant predictor (23).

Therefore, using HBM to explore further the influencing factors of the future COVID-19 vaccination intention is critical for developing targeted interventions to improve vaccine acceptance.

The theory of Planned Behavior (TPB), adapted from the theory of self-rational behavior, is one of the most influential theories for predicting and understanding whether individuals will take specific actions (24, 25). It has been widely used in the study of protection behavior during an infectious disease epidemic, such as self-isolation intention (26), H1N1 vaccination intention, and behavior (27, 28). The TPB assumes that an individual's behavioral intention is influenced by three factors: attitude toward behavior, subjective norms, and perceived behavior control, while behavioral intention further affects individual behavior (29). In the case of COVID-19 vaccination, attitude toward behavior is the positive attitude of an individual toward COVID-19 vaccination, including behavioral beliefs and evaluation of behavioral outcomes; subjective norms refer to the social pressure an individual feels in deciding whether to be vaccinated or not, for example, important others (parents, spouse, friends, colleagues) advice the individual to be vaccinated, including normative beliefs and motivation to comply; perceived behavior control is an individual's perception of the degree of control over behavior, which indicates the degree of ease or difficulty that an individual feels about COVID-19 vaccination, including perceived power and perceived behavioral control. The above three factors may affect the degree or trend of an individual's behavioral intention to vaccination (30). The TPB believes that when behavior is controlled by intention, the attitude toward behavior, subjective norms, and perceived behavioral control jointly determine an individual's behavioral intention, and perceived behavioral control can directly influence the behavior. In the context of COVID-19, Yahaghi et al. found that perceived behavioral control, subjective norms, and perceived behavior control in TPB significantly explained individuals' intention to vaccinate against COVID-19 (31), and Berg and Lin found that TPB structural variables were the essential predictors of individuals' willingness to get the COVID-19 vaccines (32).

Multiple studies have reported sociodemographic factors, health-related factors (33–37), and behavioral theories such as TPB and HBM (22, 30, 32, 38, 39) that predict the public intention to be vaccinated against COVID-19. However, during the later period of COVID-19, the public was at risk of the evolving COVID-19 variant strain and hesitated to be vaccinated against COVID-19 to a certain extent. In this context, HBM and TPB were used in this study to target populations with different COVID-19 vaccination tendencies. It compares and summarizes the relationship between vaccine hesitation/non-hesitation and the intentions to get COVID-19 vaccines and its influencing factors through different models. The study will provide scientific reference and theoretical guidance for helping health-relevant departments develop vaccination intervention plans and publicity and education programs to emerge more infectious and pathogenic variant strains.

## Materials and methods

### Participants and procedures

A national anonymous network survey was conducted using an electronic questionnaire distributed via “WJX.CN,” China's largest online survey platform, which can provide online questionnaire design and survey functions for enterprises, research institutions, and individuals. It has more than 2.6 million sample resources, helping users quickly recover accurate and effective sample data. This survey was conducted during 17–30 April 2021, when most Chinese cities were in a period of expanded vaccination coverage. The government advocated that residents eligible for COVID-19 vaccination should be vaccinated as soon as possible. This study was based on a sample database of the platform, and stratified random sampling was performed in terms of gender (1:1), age, and location; selected adults aged 18 and above who live in the Chinese mainland from the sample pool to participate in the questionnaire survey. Finally, a total of 2,098 respondents completed the survey. This study was reviewed and approved by the Peking University Institutional Review Board (IRB00001052-20081), and all respondents were informed of the survey content before signing the informed consent.

### Measures and variables

**Figure 1:**
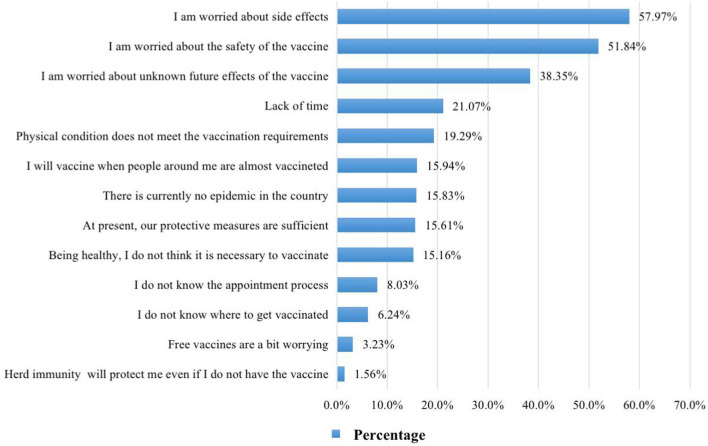
Hesitancy reason.

The dependent variable was the intention to get the COVID-19 vaccine in the future, as measured by three-item questions i,e., “If a booster of COVID-19 vaccine is required in the future, will you get vaccinated?” for measurement using the 1–5 scale (1 = strongly disagree, 5 = strongly agree). The independent variables contain four aspects. The first was basic information, including gender, age, education, marital status, and per capita monthly income. The second was the reason for vaccine hesitation, asking respondents, “What are the main reasons that you have not yet been vaccinated or delayed your vaccination?” and then select 3–5 out of the 12 alternative answers (Figure 1). The third was HBM covariables, using the Linkert 5-point scale and including perceived severity (four items assessing the individual's perception of the seriousness and consequences of COVID-19 infection), perceived susceptibility (four items assessing the individual's judgment of the likelihood of contracting COVID-19), perceived benefits (four items assessing the individual's judgment of the benefits or efficacy of receiving COVID-19 vaccine), perceived barriers (four items assessing the individual's perceived difficulties and resistance to receiving the COVID-19 vaccine), self-efficacy (four items to assess the individual's belief in their ability to perform the COVID-19 vaccination), and cues to action (including advice from family, friends, colleagues, doctors, and the government). The fourth was TPB covariables, using the Linkert 5-point scale and including (1) attitude toward behavior: behavioral beliefs (such as “Do you agree that COVID-19 vaccination can effectively reduce the risk of virus infection?”) and evaluation of behavioral outcomes (such as “Do you think it is important to reduce your own risk of COVID-19 infection?”), containing four items each in the evaluation; (2) subjective norms: normative beliefs (such as “My family thinks I should be vaccinated against Covid-19”) and motivation to comply (such as “I would like to follow the advice from those important for me to get vaccinated against COVID-19”), containing five items each; (3) perceived behavior control: control beliefs (such as “I will get vaccinated against COVID-19 even if it takes a certain amount of time and effort”) and perceived power (such as “Vaccination does not take me much time and effort”), containing two items each.

### Quality control

Quality control questions were set up in the questionnaire. It will be treated as an invalid questionnaire if the answer is not logical; questionnaires with the same answer for all options or using the same account to fill in the answers, valid questionnaires will be rewarded with gifts for thanks. Due to the online survey conducted during the particular period of COVID-19, the questionnaire content was enormous, so the effective response rate was 67.5%.

### Statistical analyses

Data processing and analysis were done using SPSS (version 26.0). Based on the WHO definition of vaccine hesitancy, the following question was used to assess the presence of vaccine hesitancy among the respondents: “Did you vaccinate at this stage?” Those who selected “vaccinated” (*n* = 860) were considered to be “Vaccine non-hesitation” For those who selected “not vaccinated” (*n* = 1,238) excluded the one selected “waiting for the unit or community arrangements” as a reason for vaccine hesitancy (*n* = 341), the remaining respondents (*n* = 897) were considered to be “Vaccine-hesitant.” Categorical variables were expressed as composition ratios, and differences in their distributions were assessed using chi-square tests. Reasons for vaccine hesitation were expressed using bar charts. The HBM and TPB covariate scores were expressed using means and standard deviations and compared between groups using *t*-tests. Backward multiple linear regression models were used to explore the factors influencing the public's intentions to receive the COVID-19 vaccine in the future. In addition, to address the issue of dimensionality in the scale and achieve homogeneity of the measurement entries, we recalculated the mean of the product sum of the corresponding entries in each of the three variables in the TPB (24, 40), at a test level of α = 0.05.

## Results

### Participant characteristics

Overall, 1757 respondents entered the final analysis. The majority are between 18 and 39 years old (85.14%), predominantly with a bachelor's degree (82.13%), married (62.78%), and about 33.30% had a per capita monthly household income of 5000–9999 (yuan). Of these, 51.05% (n=897) have vaccine hesitancy, and 48.95% do not have hesitancy. Significant differences are found between vaccine-hesitant and vaccine non-hesitant in terms of education level and marital status. People in the low education level, 66.00% have vaccine hesitancy, whereas, at the higher education level, 65.24% do not have hesitancy; the proportion of people (54.59%) with vaccine hesitancy is higher in the unmarried or single status, the proportion of people who without vaccine hesitancy is higher in the married status (51.04%) (as shown in Table 1).

**Table 1:** Characteristics of the participants (*n* = 1757).

**Characteristics**	**Total (*n* = 1757)**	**Vaccine hesitancy**		**X^2^**	***P***
		**YES (*n* = 897, 51.1%)**	**NO (*n* = 860, 48.9%)**		
Gender	Male	920 (52.36)	455 (49.46)	465 (50.54)	1.970	0.160
	Female	837 (47.64)	442 (52.81)	395 (47.19)		
Age group	18–29	757 (43.08)	404 (53.37)	353 (46.63)	6.319	0.097
	30–39	739 (42.06)	358 (48.44)	381 (51.56)		
	40–49	202 (11.50)	99 (49.01)	103 (50.99)		
	≥50	59 (3.36)	36 (51.05)	23 (48.95)		
Education level	High school and below	150 (8.54)	99 (66.00)	51 (34.00)	30.892	< 0.001
	Undergraduate	1443 (82.13)	741 (51.35)	702 (48.65)		
	Postgraduate and above	164 (9.33)	57 (34.76)	107 (65.24)		
Marital status	Unmarried	654 (37.22)	357 (54.59)	297 (45.41)	5.207	0.022
	Married	1103 (62.78)	540 (48.96)	563 (51.04)		
Monthly household income (RMB)	< 2999	125 (7.11)	72 (57.60)	53 (42.40)	5.482	0.241
	3000–4999	290 (16.51)	160 (55.17)	130 (44.83)		
	5000–9999	585 (33.30)	293 (50.09)	292 (49.91)		
	10000–14999	339 (19.29)	168 (49.56)	171 (50.44)		
	≥15000	418 (23.79)	204 (48.80)	214 (51.20)		

### Reasons for the hesitation of COVID-19 vaccine

As shown in Figure 1, concerns about the vaccine side effects (57.97%), vaccine safety (51.84%), and vaccine efficacy (38.35%) are the most important reasons for the public to hesitate about the vaccine, followed by about 20% who chose “lack of time” and “their conditions do not meet the vaccination requirements.”

### Univariate analysis

Table 2 shows the means of HBM and TPB variables for the vaccine hesitancy or not state. According to the HBM, the vaccine-hesitant group scores lower than the vaccine non-hesitant group in perceived susceptibility, perceived benefits, self-efficacy, and cues to action (including advice from family, friends, colleagues, doctors, and government) but higher than the vaccine non-hesitant group in perceived barriers. According to the TPB model, the vaccine-hesitant group scores lower than the vaccine-hesitant group on attitude toward behavior, subjective norms, and perceived behavioral control. The vaccine non-hesitant group is higher than the vaccine-hesitant group in the future vaccination intention scores.

**Table 2:** Univariate analysis between HBM and TPB variables and the hesitation to vaccinate with COVID-19.

	**Variables**	**Vaccine hesitancy**		***t***	***P***
		**YES (*n* = 897)**	**NO (*n* = 860)**		
HBM	Perceived severity	4.290 ± .66	4.620 ± .44	– 1.742	0.082
	Perceived susceptibility	4.280 ± .67	4.220 ± .772	– 2.511	0.012
	Perceived benefits	4.120 ± .57	4.310 ± .50	7.274	< 0.001
	Perceived barriers	2.400 ± .71	2.000 ± .63	– 12.365	< 0.001
	Self-efficacy	3.710 ± .83	4.250 ± .57	15.894	< 0.001
	Advice from family	3.870 ± .86	4.320 ± .69	12.017	< 0.001
	Advice from friends	3.600 ± .89	3.970 ± .80	9.084	< 0.001
	Advice from colleagues	3.810 ± .93	4.200 ± .80	9.503	< 0.001
	Advice from doctors	4.070 ± .86	4.240 ± .76	4.419	< 0.001
	Advice from government	4.220 ± .78	4.300 ± .76	2.035	0.042
TPB	Attitude toward behavior	4.320 ± .44	4.450 ± .38	6.508	< 0.001
	Subjective norms	3.930 ± .61	4.220 ± .47	11.369	< 0.001
	Perceived behavioral control	3.440 ± .74	3.890 ± .65	13.431	< 0.001
	Future vaccination intention	4.290 ± .66	4.620 ± .44	12.328	< 0.001

HBM, Health Belief Model; TPB, Theory of Planned Behavior.

### Multivariate analysis

As shown in Table 3, the acceptance of future vaccines in the vaccine-hesitant group was explored based on HBM and TPB. Model 1, which includes sociodemographic variables, the health-related factor, and HBM variables, explains 42.3% of the vaccine-hesitant group's intention to receive the future COVID-19 vaccine. According to the model, younger individuals (β = – 0.107, *P* < 0.001), more excellent vaccine knowledge (β = 0.032, *P* = 0.002), perceived benefits (β = 0.122, *P* < 0.001), self-efficacy (β = 0.241, *P* < 0.001) and advice from family (β = 0.155, *P* < 0.001) and doctors (β = 0.099, *P* < 0.001) are significant predictors of future vaccination intentions in the vaccine-hesitant group. Model 2 includes sociodemographic variables, vaccine knowledge, and TPB variables, which explains 46.4% of future COVID-19 vaccination intentions. In Model 2, age (β = – 0.094, *P* < 0.001), vaccine knowledge (β = 0.020, *P* = 0.043), attitude toward behavior (β = 0.189, *P* < 0.001), subjective norms (β = 0.580, *P* < 0.001), perceived behavioral control (β = 0.241, *P* < 0.001) are essential predictors of the future vaccination willingness of vaccine hesitating groups.

**Table 3:** Multiple linear regression analysis - predictors of intention to get future vaccinated against COVID-19 (the vaccine-hesitant group).

	**Model 1: block1,2,3**				**Model 2: block1,2,4**			
	**β**	**Beta**	**95%CI of β**	***P***	**β**	**Beta**	**95%CI of β**	***P***
Constant	1.401		(1.073,1.728)	< 0.001	0.869		(0.518,1.220)	< 0.001
**Block1:Sociodemographic**								
Age	–0.107	–0.132	(–0.148,–0.066)	< 0.001	–0.094	–0.116	(–0.133,–0.054)	< 0.001
Education level								
**Block2:Health-related factor**								
Vaccine knowledge	0.032	0.081	(0.012,0.051)	0.002	0.020	0.051	(0.001,0.039)	0.043
**Block3:HBM**								
Perceived severity								
Perceived susceptibility								
Perceived benefits	0.122	0.106	(0.055,0.189)	< 0.001				
Perceived barriers								
Self-efficacy	0.241	0.305	(0.191,0.291)	< 0.001				
Advice from family	0.155	0.204	(0.105,0.205)	< 0.001				
Advice from friends								
Advice from colleagues								
Advice from doctors	0.099	0.130	(0.054,0.144)	< 0.001				
Advice from government								
**Block4:TPB**								
Attitude toward behavior					0.189	0.158	(0.103,0.275)	< 0.001
Subjective norms					0.580	0.502	(0.515,0.644)	< 0.001
Perceived behavioral control					0.077	0.092	(0.028,0.126)	0.002
R^2^	0.423		0.464					

HBM, Health Belief Model; TPB, Theory of Planned Behavior.

In Table 4, we used HBM and TPB to explore the vaccination intentions of the vaccine non-hesitant group for the future COVID-19 vaccine. According to Model 3, education level (β = 0.059, *P* = 0.045), marriage (β = – 0.096, *P* = 0.004), perception Behavior benefits (β = 0.094, *P* = 0.002), perceived behavior disorder (β = – 0.062, *P* = 0.006), self-efficacy (β = 0.273, *P* < 0.001), advice from family (β = 0.046, *P* = 0.023) and doctors (β = 0.065, *P* = 0.001) are essential factors influencing the intention of future vaccination with an explanation level of 36.0%. Model 4 explains 33.0% of future COVID-19 vaccination intentions, age (β = 0.041, *P* = 0.047), marital status (β = – 0.086, *P* = 0.010), attitude (β = 0.341, *P* < 0.001), subjective behavioral norms (β = 0.250, *P* < 0.001) and perceptual behavior control (β = 0.072, *P* = 0.001) are important predictors of the future vaccination willingness of the vaccine unhesitating group.

**Table 4:** Multiple linear regression analysis–predictors of intention to get future vaccinated against COVID-19 (the vaccine non-hesitant group).

	**Mode 3: block1,2,3**				**Model 4: block1,2,4**			
	**β**	**Beta**	**95%CI of β**	***P***	**β**	**Beta**	**95%CI of β**	***P***
Constant	2.285		(1.891,2.678)	< 0.001	1.533		(1.185,1.881)	< 0.001
**Block1:Sociodemographic**								
Age					0.041	0.071	(0.001,0.082)	0.047
Education level	0.059	0.056	(0.001,0.116)	0.045				
Marital status	–0.096	–0.103	(–0.161,–0.032)	0.004	–0.086	–0.092	(–0.151,–0.021)	0.010
**Block2:Health-related factor**								
Vaccine knowledge								
**Block3:HBM**								
Perceived severity								
Perceived susceptibility								
Perceived benefits	0.094	0.105	(0.034,0.153)	0.002				
Perceived barriers	–0.062	–0.087	(–0.106,–0.018)	0.006				
Self-efficacy	0.273	0.351	(0.217,0.328)	< 0.001				
Advice from family	0.046	0.072	(0.006,0.085)	0.023				
Advice from friends								
Advice from colleagues								
Advice from doctors	0.065	0.113	(0.028,0.103)	0.001				
Advice from government								
**Block4:TPB**								
Attitude toward behavior					0.341	0.296	(0.259,0.423)	< 0.001
Subjective norms					0.250	0.267	(0.185,0.316)	< 0.001
Perceived behavioral control					0.072	0.107	(0.028,0.117)	0.001
R^2^	0.360		0.330					

HBM, Health Belief Model; TPB, Theory of Planned Behavior.

## Discussion

During the investigation of this study, China's COVID-19 vaccine program had already been available to the general public, and the government had actively called for eligible individuals to be vaccinated against COVID-19 as soon as possible. This study aims at people with different COVID-19 vaccination tendencies, using HBM and TPB models to understand the relationship between vaccine hesitation/non-hesitation and the intentions to get COVID-19 vaccines and its intentions influencing factors from different aspects but also to compare and summarize the model results. The obtained results are of specific reference significance for developing the future vaccination plan and publicity and education work of health-related departments.

This study found that educational background is one of the influencing factors for vaccine hesitation. Most people with high education do not hesitate, while a larger proportion of people with low education have vaccine hesitation. Secondly, concerns about the safety, side effects, and efficacy of the COVID-19 vaccine are the leading cause of public vaccine hesitation in this study, similar to the results of Robertson (41) studying vaccine hesitation in the UK general population. Compared with other vaccines, the speed of the COVID-19 vaccine from research and development and clinical trials to coming into use is unprecedented (42–44). The COVID-19 virus has constantly been evolving, and the durability of immune response after vaccination and the vaccine's effectiveness for transmission have not been answered in clinical trials, which may affect public acceptance of the COVID-19 vaccine. Thirdly, in the context of COVID-19, vaccine hesitation will still affect future vaccination intentions. According to HBM, perceived severity, perceived susceptibility, perceived benefits and barriers, and self-efficacy are essential predictors of intention to receive the COVID-19 vaccine. In contrast, the HBM variable scores are higher in the population without vaccine hesitation (except for perceived behavioral disorder) than those with vaccine hesitation. According to TPB, the more positive the attitude toward vaccines (45–47), the higher the pressure from others or society (48), and the higher the control ability (49, 50), the higher the acceptance of vaccination behavior. The results found that the vaccine non-hesitation group in attitude, subjective norms, and perceived behavioral control scores were higher than the vaccine hesitation group.

This study helps to understand further the influencing factors of future vaccination intentions among populations with different COVID-19 vaccination tendencies through HBM and TPB. According to models 1 and 3, in sociodemographic and health-related factors, the vaccine hesitation group is affected by age and vaccine knowledge, which indicates that younger people are more receptive than the older, and vaccine knowledge is a factor in improving future generations' COVID-19 vaccination intention. The vaccine non-hesitation group is affected by educational level and marital status, indicating that more educated people, unmarried or single, are more likely to receive COVID-19 vaccination. According to HBM, the common point of the two groups (vaccine hesitation/non-hesitation) is that the future vaccination intention is all affected by perceived benefits, self-efficacy, advice from family and doctors, and self-efficacy plays the most significant role. The difference is that for the vaccine hesitation group, in addition to improving vaccination confidence (self-efficacy), advice from family and doctors could also help improve future vaccination intention; for the vaccine non-hesitation group, in addition to improving self-efficacy and listening to doctors' advice, the positive attitude toward the future vaccination can also be enhanced by raising awareness of the benefits and efficacy of the COVID-19 vaccine, which can indicate that self-efficacy is an important influencing factor of future vaccination intentions through HBM. Promoting the public's vaccination intentions by publicizing the benefits of vaccines and making recommendations through family members and doctors is of great significance. Similar to previous studies (22, 23, 32), this study used HBM to predict the acceptance of the COVID-19 vaccine, finding that the public pays more attention to the vaccine's efficacy, safety, and cost than to disease severity or susceptibility.

According to models 2 and 4, the two groups (vaccine hesitation/non-hesitation) are affected by age. However, the difference is that younger people have higher vaccination intentions in the vaccine hesitation group, while in the vaccine non-hesitation group, older people have higher vaccination intentions. According to TPB, the future vaccination intention is affected by three factors: attitude, subjective norms, and perceived behavioral control. However, in the vaccine hesitation group, subjective norms are the main influencing factor, consistent with Berg's findings that subjective behavioral norms significantly predict vaccine intentions, further proving that vaccine-hesitant groups usually need more encouragement and establishment from people around them to increase vaccination intentions. In the vaccine non-hesitation group, the attitude toward behavior is the main factor in improving behavioral intention.

In models 1 and 2, HBM and TPB were used to predict the influencing factors of the future vaccination intention in vaccine hesitation groups, respectively. In HBM, self-efficacy, and advice from family and doctors significantly influence the future vaccination intention, while subjective norms are the main influencing factor in TPB. In theory, subjective norms refer to the pressure individuals feel from important others (parents, friends, colleagues, doctors) when deciding whether or not to receive the COVID-19 vaccine. Important others think that they should be vaccinated, and individuals also follow the advice from “others.” Therefore, for the vaccine hesitation group, self-confidence and social pressure are the main factors affecting future vaccination intention. Models 3 and 4 show that in HBM, self-efficacy, perceived benefits, and medical advice significantly influence future vaccination intention. In TPB, the attitude toward behavior is the most significant influencing factor, theoretically referring to the positive attitude of individuals to the COVID-19 vaccination and the evaluation of vaccine effectiveness. Therefore, the benefits and effects of vaccination are important factors of the future vaccination intention for such groups. According to TPB, attitude toward behavior is assessed by behavioral beliefs and behavioral outcomes. Behavioral beliefs are similar to the concept of perceived benefits in HBM, so it is further demonstrated through HBM and TPB that recommendation from others is critical for increasing vaccination intentions among the hesitant-vaccine group. For the group who are not hesitant to vaccinate, both HBM and TPB expressed their intention to increase vaccination intentions by raising awareness of the benefits of vaccination.

This study demonstrated that public vaccine hesitation does impact future vaccination intentions in the latter period of the COVID-19 pandemic. As a result, it is suggested that future intervention plans should deal with vaccine hesitation and lower willingness to receive the future vaccination to ensure the proportion of actual vaccination, especially in high-risk groups. Specifically, in the future vaccine plan, it can be considered whether there is hesitation about the “current” vaccine. Since most people with vaccine hesitation are less educated, single, or unmarried, public health interventions should focus on vaccine-related knowledge and improve the confidence and ability of vaccination. In addition, consideration should also be given to relatives and doctors who share positive ideas and experiences of the COVID-19 vaccination, such as encouraging them to share the time, location, and feelings of vaccination on community social platforms or family chat groups. However, most people with vaccine non-hesitation are highly educated and married and usually have a certain sense of vaccination. Due to the family's responsibility, learning ability, and accessibility to learning resources, they may further explore related information about the safety, effectiveness, and efficacy of the vaccine to protect the life safety of themselves and their families. Therefore, for these people, future public health interventions should increase awareness of vaccination benefits, provide more evidence on the safety and efficacy of the COVID-19 vaccine, and highlight the vaccine benefits for individuals and groups.

Furthermore, both HBM and TPB have a certain degree of interpretation for the vaccination intention in this study, where TPB is higher for the vaccine hesitation group. However, HBM is higher for the vaccine non-hesitation group. Therefore, based on the premise of public health intervention workload, the design of intervention or health education plans for different groups (such as vaccine hesitation) on the theoretical basis of different health behavior models can also be used as one of the alternatives for future intervention.

This study has several limitations that should be recognized when interpreting the results reported here. First of all, like other surveys in pandemics, this study used online survey platforms to conduct online surveys, and the sample size may limit the representativeness of the results. However, to solve this problem, we increased the sample size and random stratified sampling based on gender, age, and location. Secondly, the sample exclusion criteria of this study include people who do not use smartphones or fill out electronic questionnaires. Most of them are older people over 50 years old, which will lack the representativeness of this age group. Third, the study mainly predicts future COVID-19 vaccination intentions through self-reports which may have deviations from the objective measurement of actual vaccination in the future. We will continue to track the same group to obtain objective measurement data of actual vaccination in the future.

## Conclusions

First, in the context of COVID-19, the public's hesitation on the “current” vaccines will still affect future vaccination intentions. Secondly, HBM and TPB models can help health policymakers and healthcare providers formulate intervention plans. Specifically, for vaccine hesitation groups, it is recommended to improve vaccine-related knowledge and through the ways of persuasion by others (family members, doctors) to enhance individual's willingness to acceptance vaccinate in the future; for those who do not hesitate about vaccines, raise awareness of the benefits of vaccination, provide more evidence on the safety and effectiveness of the COVID-19 vaccine to increase public vaccination intentions.

## Data availability statement

The data analyzed in this study is subject to the following licenses/restrictions: The data that support the finding of this study are available on request from the corresponding author. The data are not publicly available due to privacy or ethical restrictions. Requests to access these datasets should be directed to xysun@bjmu.edu.cn.

## Ethics statement

The studies involving human participants were reviewed and approved by the Institutional Review Board of Peking University. Written informed consent for participation was not required for this study in accordance with the national legislation and the institutional requirements.

## Author contributions

ZL: conceptualization, methodology, software, and writing—original draft preparation. XS: review and editing, supervision, and project administration. YJ: revision of the manuscript. All authors contributed to the article and approved the submitted version.

## Funding

This work was supported by the National Social Science Fund of China (22AZD077).

## Conflict of interest

The authors declare that the research was conducted in the absence of any commercial or financial relationships that could be construed as a potential conflict of interest.

## Publisher's note

All claims expressed in this article are solely those of the authors and do not necessarily represent those of their affiliated organizations, or those of the publisher, the editors and the reviewers. Any product that may be evaluated in this article, or claim that may be made by its manufacturer, is not guaranteed or endorsed by the publisher.
