# Supplementary material for: The impact of vaccine hesitation on the intentions to get COVID-19 vaccines: The use of the health belief model and the theory of planned behavior model
Source: Front Public Health. 2022 Oct 14;10:882909. doi: 10.3389/fpubh.2022.882909 (PMC9615565; doi:10.3389/fpubh.2022.882909)
Supplement: Supplementary file 1 [file Table_1.docx]

Measures for HBM used in this study are listed in Table 1.

| Table 1. Measures for HBM used in the present study. | | |
| --- | --- | --- |
| Measures | Items | Response Scale |
| Perceived Severity | Item 1:Infection with COVID-19 would cause serious health problems. | 1 (strongly disagree) to 5 (strongly agree) |
|  | Item 2: Infection with COVID-19 would have a detrimental effect on mental health, leading to anxiety, fear, depression, and other negative emotions. |  |
|  | Item 3: Infection with COVID-19 would have a severe impact on daily life. |  |
|  | Item 4: Infection with COVID-19 would affect one's or a family's financial income. |  |
| Perceived Susceptibility | Item 1: Possibility of acquiring COVID-19 when studying and working in the same space as an infected person. | 1 (not at all) to 5 (certain) |
|  | Item 2: Possibility of acquiring COVID-19 if you use the same indoor air purification system as an infected person. |  |
|  | Item 3: Possibility of living in the same unit building as the infected person. |  |
|  | Item 4: Possibility of taking the same transportation as the infected person. |  |
| Perceived Benefits | Item 1:Vaccination is a very effective way to protect you against COVID-19. | 1 (strongly disagree) to 5 (strongly agree) |
|  | Item 2:Vaccination greatly reduces the risk of infection to your family and others around you. |  |
|  | Item 3:Vaccination helps you to concentrate more on your studies, work, and life. |  |
|  | Item 4:Vaccination helps to end the epidemic as soon as possible. |  |
| Perceived Barriers | Item 1:Safety and possible side effects of vaccine. | 1 (strongly disagree) to 5 (strongly agree) |
|  | Item 2:Vaccination can be psychologically taxing. |  |
|  | Item 3:Vaccine is also a virus will increase the risk of infection. |  |
|  | Item 4:Vaccination will take time and effort. |  |
| Self-efficacy | Item 1:I will be vaccinated even if I test negative for COVID-19. | 1 (strongly disagree) to 5 (strongly agree) |
|  | Item 2:I will be vaccinated even if there are no new confirmed cases in my city. |  |
|  | Item 3:I will get vaccinated even if people around me think it is unnecessary. |  |
|  | Item 4:I will get vaccinated even if the vaccination facility is far from me. |  |
| Cues to action | Item 1: My family thinks I should be vaccinated against Covid-19. | 1 (strongly disagree) to 5 (strongly agree) |
|  | Item 2: People around me (friends, colleagues, classmates, neighbors, etc.) think I should be vaccinated against the COVID-19. |  |
|  | Item 3: The superior leader of the unit or company suggested that I should be vaccinated against the COVID-19. |  |
|  | Item 4: The community doctor suggested that I should be vaccinated against the COVID-19. |  |
|  | Item 5: The staff of the health administrative department suggested that I should be vaccinated against the COVID-19. |  |

Measures for TPB used in this study are listed in Table 2.

| Table 2. Measures for TPB used in the present study. | | |
| --- | --- | --- |
| Measures | Items | Response Scale |
| Behavioral Beliefs | Item A1: Do you agree that, vaccination is a very effective way to protect you against COVID-19. | 1 (strongly disagree) to 5 (strongly agree) |
|  | Item A2: Do you agree that, vaccination greatly reduces the risk of infection to your family and others around you. |  |
|  | Item A3: Do you agree that, vaccination could help you to concentrate more on your studies, work, and life. |  |
|  | Item A4: Do you agree that, vaccination helps to end the epidemic as soon as possible. |  |
| Behavioral Outcomes | Item B1: Do you think it is important to reduce your risk of contracting the COVID-19 ? | 1 (not at all) to 5 (certain) |
|  | Item B2: Do you think it is important to reduce the risk of infection with the COVID-19 in your family and others around you? |  |
|  | Item B3: Do you think it is important to be able to devote yourself to study, work and life? |  |
|  | Item B4: Do you think it is important to end the epidemic as soon as possible? |  |
| Normative Beliefs | Item 1: My family thinks I should be vaccinated against Covid-19. | 1 (strongly disagree) to 5 (strongly agree) |
|  | Item 2: People around me (friends, colleagues, classmates, neighbors, etc.) think I should be vaccinated against the COVID-19. |  |
|  | Item 3: The superior leader of the unit or company suggested that I should be vaccinated against the COVID-19. |  |
|  | Item 4: The community doctor suggested that I should be vaccinated against the COVID-19. |  |
|  | Item 5: The staff of the health administrative department suggested that I should be vaccinated against the COVID-19. |  |
| Motivation to Comply | Item 1: I would like to follow the advice from my family to get vaccinated. | 1 (strongly disagree) to 5 (strongly agree) |
|  | Item 2: I would like to follow the advice from People around me (friends, colleagues, classmates, neighbors, etc.) to get vaccinated. |  |
|  | Item 3: I would like to follow the advice from the superior leader of the unit or company to get vaccinated. |  |
|  | Item 4: I would like to follow the advice from doctor to get vaccinated. |  |
|  | Item 5: I would like to follow the advice from the staff of the health administrative department to get vaccinated. |  |
| Control Beliefs | Item 1: I will get vaccinated against COVID-19 even if it takes a certain amount of time and effort. | 1 (strongly disagree) to 5 (strongly agree) |
|  | Item 2: I will get vaccinated against COVID-19 even if Even if I am busy with study and work. |  |
| Perceived Power | Item 1: Getting vaccinated doesn’t take much time and effort. | 1 (strongly disagree) to 5 (strongly agree) |
|  | Item 2: It will not take too much time and effort to acquire and learn knowledge about the new crown vaccine. |  |

Measures for the intention to get COVID-19 vaccine used in this study are listed in Table 3.

| Table 3. Measures the intention to get COVID-19 vaccine used in the present study. | | |
| --- | --- | --- |
| Measures | Items | Response Scale |
| Perceived Severity | Item 1:If a booster of COVID-19 vaccine is required in the future, will you get vaccinated? | 1 (strongly disagree) to 5 (strongly agree) |
|  | Item 2: If the variants of the COVID-19 requires a "new" vaccine, and the vaccine is proven to be safe and effective, and the vaccination service is available, will you be vaccinated in the future? |  |
|  | Item 3: If the variants of the COVID-19 requires a "new" vaccine, the government recommends vaccination, will you be vaccinated in the future? |  |
